# 868 Beyond Resuscitation: Early Vasopressor Use as an Indicator of Clinical Severity in Burn Care Outcomes

**DOI:** 10.1093/jbcr/iraf019.399

**Published:** 2025-04-01

**Authors:** Cole Bird, Sofie Hass, Ragan Verelst, Fatma Ulusan Sayali, Jessica Reynolds, Niaman Nazir, Dhaval Bhavsar

**Affiliations:** University of Kansas School of Medicine; University of Kansas School of Medicine; University of Kansas Medical Center; University of Kansas Medical Center; The University of Kansas Health System; University of Kansas Medical Center; The University of Kansas Health System Burnett Burn Center

## Abstract

**Introduction:**

Vasopressors are often necessary to manage hypotension during early shock in critically ill burn patients. This study examines the early use of vasopressors—administered within the first 72 hours post-burn injury—and its use as an indicator for key clinical outcomes.

**Methods:**

We performed a retrospective chart review of adult burn patients (≥18 years) admitted to a regional burn center between 2010 and 2023. Patients included in the study had burns covering ≥20% total body surface area (TBSA) and received vasopressors within 72 hours of burn injury and continued for at least 6 hrs. Those who received vasopressors formed the VASO group, while patients not receiving vasopressors comprised the control group. Outcomes of interest, such as length of hospital stay, number of surgical interventions, and mortality, were compared between 17 VASO and over 100 control patients matched by age, race, and TBSA%. Statistical analyses included chi-square tests for categorical variables, t-tests for continuous variables, and multivariate regression to adjust for confounders.

**Results:**

The VASO and control groups were similar in age (45.9 vs. 40.5, p = 0.15), race (p = 0.73), and TBSA% (43.1 vs. 34.1, p = 0.13), respectively. However, the VASO group patients had significantly longer hospital stays (85.18 vs. 30.32, p = 0.012) and required more surgical interventions (12.41 vs. 3.96, p = 0.004) compared to control patients. The VASO group also had a greater hospital stay per percentage TBSA burned (42.01 vs -2.38, p = 0.012), indicating increased clinical severity. Mortality rate was not different between the groups.

**Conclusions:**

Early vasopressor use during burn resuscitation correlates with increased clinical severity, as reflected in extended hospital stays, greater surgical needs. These findings suggest vasopressor administration may serve as a useful indicator of disease severity, warranting further investigation into its role in optimizing burn resuscitation protocols.

**Applicability of Research to Practice:**

This study identifies early vasopressor use as a marker of increased clinical severity in burn patients, potentially aiding clinicians in assessing patient condition and tailoring resuscitation strategies. Integrating vasopressor use into clinical decision-making could enhance resource allocation, improve patient outcomes, and inform future burn care protocols. Further research, including prospective studies, is recommended to clarify the benefits and risks associated with vasopressor use in burn resuscitation.

**Funding for the Study:**

N/A

